# Treatment of epilepsy using a targeted p38γ kinase gene therapy

**DOI:** 10.1126/sciadv.add2577

**Published:** 2022-12-02

**Authors:** Nicolle Morey, Magdalena Przybyla, Julia van der Hoven, Yazi D. Ke, Fabien Delerue, Janet van Eersel, Lars M. Ittner

**Affiliations:** Dementia Research Centre, Macquarie Medical School, Faculty of Medicine, Health and Human Sciences, Macquarie University, Sydney, Australia.

## Abstract

Hyperphosphorylated microtubule-associated protein tau has been implicated in dementia, epilepsy, and other neurological disorders. In contrast, site-specific phosphorylation of tau at threonine 205 (T205) by the kinase p38γ was shown to disengage tau from toxic pathways, serving a neuroprotective function in Alzheimer’s disease. Using a viral-mediated gene delivery approach in different mouse models of epilepsy, we show that p38γ activity–enhancing treatment reduces seizure susceptibility, restores neuronal firing patterns, reduces behavioral deficits, and ameliorates epilepsy-induced deaths. Furthermore, we show that p38γ-mediated phosphorylation of tau at T205 is essential for this protection in epilepsy, as a lack of this critical interaction reinstates pathological features and accelerates epilepsy in vivo. Hence, our work provides a scope to harness p38γ as a future therapy applicable to acute neurological conditions.

## Teaser

Gene delivery into epilepsy mouse models reveals a therapeutic role for tau modulation in drug-resistant seizure disorders.

## INTRODUCTION

Epilepsy currently affects 70 million people globally and is among the most prevalent neurological diseases (*1*). Epilepsy can emerge from genetic or acquired etiologies; however, neuronal network disruption commonly underpins epileptogenesis. Accordingly, excessive excitatory neuronal stimulation and impaired inhibitory function trigger hyperexcitation and cellular toxicity (=excitotoxicity), a shared feature of a broad range of neurological disorders including dementia, stroke, and epilepsy. In the case of pharmacoresistant epilepsies, this network dysfunction drives progressive clinical phenotypes, including depression and other psychiatric disorders, cognitive impairment, and epilepsy-related mortality (*2*, *3*). Given that patients with dementia and stroke patients experience higher seizure incidence and patients with certain epilepsy disorders are at a greater risk of developing dementia and stroke (*4*, *5*), intervening therapies targeting a common implicative factor are of great interest. Research in Alzheimer’s disease (AD) by us and others has implicated the tau protein in excitotoxic signaling (*6*). Accordingly, neuronal hyperexcitation engages tau-dependent postsynaptic excitotoxic signaling complexes that drive downstream clinical phenotypes, and this process is augmented by accrual of hyperphosphorylated tau at the postsynapse. Furthermore, tau is implicated as a propeller of excitotoxicity in stroke, autism, and epilepsy syndromes (*7*, *8*), suggesting a converging, tau-dependent pathomechanism across neurological disorders. Although tau hyperphosphorylation characterizes its pathological state, we have shown that phosphorylation of tau by the p38 mitogen-activated protein kinase (MAPK), p38γ, imparts neuroprotection from downstream excitotoxicity in AD mouse models (*9*, *10*). Here, postsynaptic p38γ mediates protection through its kinase activity at tau residue threonine 205 (tauT205), which disengages tau from toxic signaling complex formation, in turn reducing neurotoxicity and alleviating cognitive deterioration (*9*, *10*). To probe the mechanistic relevance of this postsynaptic tau/p38γ interaction in epilepsy, we generated two epilepsy mouse models: a genetic model of the infantile-onset epileptic encephalopathy Dravet syndrome (DS) and a chemically induced epilepsy mouse model to study acute and chronic seizure effects. In both cases, tau is a known mediator of neurotoxicity; genetic tau depletion in both DS and chemically induced seizure mice imparts seizure prevention (*11*–*13*). Nonetheless, the exact mechanisms by which tau contributes to epilepsy remain largely unknown. Here, we demonstrate significant antiepileptic effects in both epilepsy models following induced p38γ activity and show mechanistically that this kinase imparts its protection through site-specific interaction with tau.

## RESULTS

### p38γ kinase treatment protects DS mice

**Fig. 1.**
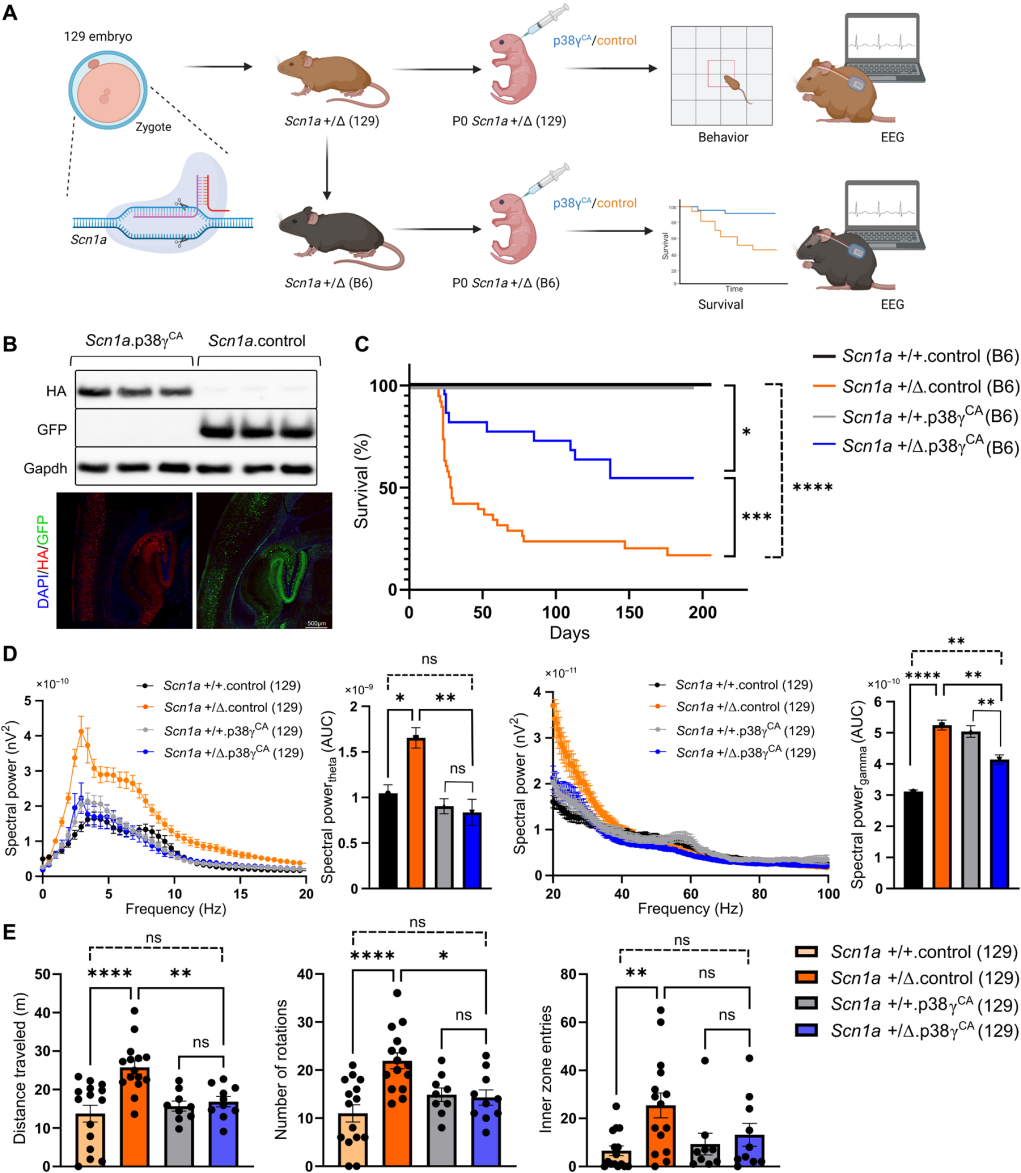
p38γ kinase treatment protects DS mice. (**A**) *Scn1a* +/Δ mouse line generation and experimental strategy for p38γ therapeutic study. (**B**) Western blotting of the cortex (top) and immunostaining of the hippocampus (bottom) validated the transgene expression of p38γ^CA^ [hemagglutinin (HA) tagged] or control [green fluorescent protein (GFP)] in 6-month-old *Scn1a* (B6) mice (representative images from *n* = 4 to 6 mice per treatment). Glyceraldehyde-3 phosphate (GAPDH) served as a loading control. DAPI, 4′,6-diamidino-2-phenylindole. (**C**) Survival curve for treated *Scn1a* (B6) mice; *Scn1a* +/Δ.p38γ^CA^ (B6) mice show significantly improved survival compared with nontherapeutic control-administered mice [*Scn1a* +/Δ.control (B6)] over 200 days [*n* ≥ 11 per group; *P = 0.0113, ***P = 0.0006, and ****P ≤ 0.0001 (Mantel-Cox test)]. (**D**) Electroencephalographic (EEG) power spectral density profiles at low (left) and high (right) frequencies during wakeful periods in 5- to 6-month-old treated Scn1a (129) mice. *Scn1a* +/Δ.control (129) mice demonstrated significantly elevated power spectral density when compared with *Scn1a* +/+ mice in theta (4- to 12-Hz) and gamma (25- to 100-Hz) frequency bands [*P = 0.0156 (theta) and ****P ≤ 0.0001 (gamma)]. Aberrant network activity was ameliorated in *Scn1a* +/Δ.p38γ^CA^ (129) mice that exhibited theta power not significantly different from that of *Scn1a* +/+ mice of both treatment groups [**P = 0.002 (theta) and **P = 0.0016 (gamma); ns, not significant (ANOVA)], although gamma power remained moderately increased compared with *Scn1a* +/+.control mice (**P = 0.0015). Gamma power in *Scn1a* +/+.p38γ^CA^ was significantly altered compared to *Scn1a* +/+.control and *Scn1a* +/+.p38γ^CA^ mice (*n* = 3 to 5 mice per group; ****P ≤ 0.0001 and **P = 0.0041). AUC, area under the curve. (**E**) Open-field behavioral analysis. Compared with *Scn1a* +/+.control (129) mice, *Scn1a* +/Δ.control (129) mice traveled significantly further distances, with more circling, and more frequently in inner zone entries [****P ≤ 0.0001 (distance), ****P ≤ 0.0001 (circling), and **P = 0.0012 (zone)]. Behaviors of *Scn1a* +/Δ.p38γ^CA^ (129) mice were significantly normalized compared with *Scn1a* +/Δ.control (129) mice [**P = 0.0086 (distance), *P = 0.0164 (circling), and ns, *P* = 0.2012 (zone)] and not significantly different from that of *Scn1a* +/+ mice of both treatment groups [*n* = 9 to 15 mice per group; ns (ANOVA)].

Among the most severe childhood epilepsies, DS predominantly emerges from heterozygous, loss-of-function pathogenic variants in the *SCN1A* gene, which encodes a sodium channel subunit required for inhibitory interneuron function. As a result, neuronal hyperexcitation triggers intractable seizures, behavioral disturbances, and high rates of sudden unexpected death in epilepsy (SUDEP) in patients (*14*). To study this encephalopathy, an *Scn1a* loss-of-function mouse model of DS was generated by targeting exon 1 of *Scn1a* in a 129 background strain, termed *Scn1a* (129; Fig. 1A and fig. S1A). Consistent with previous reports (*15*), all homozygous *Scn1a* Δ/Δ (129) mice (harboring complete *Scn1a* loss of function) exhibited ataxia, weight loss, and seizures from postnatal day 10 (P10) and seizure-induced lethality by 2 weeks of age (fig. S1B). Heterozygous *Scn1a* +/Δ (129) mice presented with normal life expectancy and overt seizures were absent; however, they demonstrated neuronal network abnormalities and behaviors consistent with DS (fig. S1, B to E). In further congruence with previous reports (*15*), when crossed onto the seizure-susceptible C57Bl/6 (B6) background, from 3 weeks of age, a high proportion of *Scn1a* +/Δ (B6) mice exhibited aberrant behaviors and overt seizures shortly before SUDEP (fig. S1, F to H). Thus, use of *Scn1a* +/Δ mice on both 129 and B6 backgrounds enabled the study of different DS aspects. To assess the therapeutic impact of p38γ on DS phenotypes, an adeno-associated viral (AAV)–mediated gene delivery approach was used for neuron-specific expression (see Materials and Methods). At birth (P0), *Scn1a* mice on both backgrounds were administered neurotropic AAV intravenously (Fig. 1A) for neuronal expression of either a constitutively active (CA) form of p38γ (*Scn1a*.p38γ^CA^) or a nontherapeutic control (*Scn1a.*control), and their expression was confirmed by Western blot and immunostaining (Fig. 1B). To assess the therapeutic impact of p38γ on survival, *Scn1a* (B6) mice were monitored for rates of seizure-induced mortality over a 7-month period; notably, the incidence of premature mortality was significantly reduced in *Scn1a* +/Δ.p38γ^CA^ (B6) mice, compared to *Scn1a* +/Δ.control (B6) mice (Fig. 1C). No differences in survival between male and female mice within each treatment group were found.

Hippocampal electroencephalographic (EEG) recordings show the activity of local neuronal populations, with theta (4- to 12-Hz) and gamma (25- to 100-Hz) oscillation patterns (generated by firing of excitatory and inhibitory neurons, respectively) indicating the extent of neuronal network synchronicity and cognitive function (*16*). While hippocampal EEG recordings of *Scn1a* +/Δ.control mice on both 129 and B6 backgrounds did not exhibit spontaneous epileptiform discharges or seizure episodes during the recording period (fig. S1C), their EEGs were presented with significantly abnormal theta and gamma spectral power during wake phases compared to wild-type littermates (Fig. 1D and fig. S2A). The elevated power in the theta range represents insufficient inhibition, consistent with aberrant low-frequency oscillations reported in patients with DS and DS mouse models (*17*), while elevated gamma power indicates a compensatory response to combat aberrantly raised theta power. In contrast, treated *Scn1a* +/Δ.p38γ^CA^ mice on both 129 and B6 backgrounds exhibited normalized spectral power in both theta and gamma frequencies (Fig. 1D and fig. S2A). Given the high mortality of *Scn1a* +/Δ.control (B6) mice (Fig. 1B and fig. S2, B and C), to avoid mortality bias, *Scn1a* (129) mice were used to assess the therapeutic impact of p38γ on DS behavioral phenotypes. To assess locomotor behaviors, *Scn1a* (129) mice were tested in the open-field test paradigm (see Materials and Methods). Consistent with hyperactivity and autism-like traits in patients with DS, *Scn1a* +/Δ.control (129) mice displayed hyperactivity with significantly greater distances traveled, more circling behavior, and a preference to enter the arena center when compared to wild-type *Scn1a* +/+ (129) mice from both treatment groups (Fig. 1E). Conversely, reduced aberrant behaviors of treated *Scn1a* +/Δ.p38γ^CA^ mice emerged, compared to *Scn1a* +/Δ.control (129) mice. In addition, behaviors of *Scn1a* +/Δ.p38γ^CA^ (129) mice did not differ significantly from that of wild-type mice administered with either control or p38γ^CA^ AAV (Fig. 1E). No differences in behavior between male and female mice within each treatment group were found.

### TauT205 is a protective site in epilepsy

**Fig. 2.**
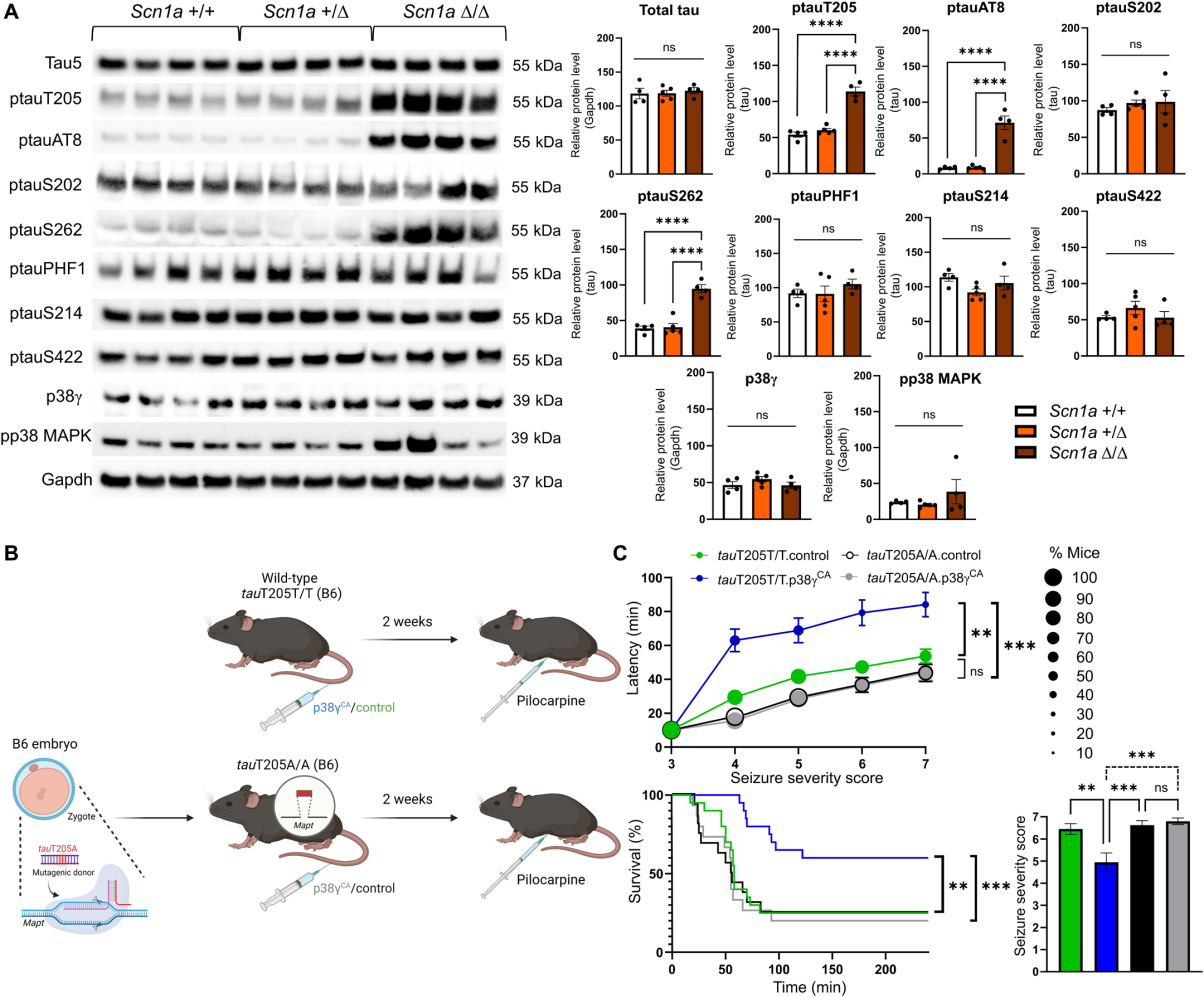
TauT205 is a protective site in epilepsy. (**A**) Western blotting of brains of 2-week-old *Scn1a* Δ/Δ (129) mice shows significantly increased and distinct tau phosphorylation at T205 (ptauT205), S202 + T205 (ptauAT8), and S262 (ptauS262) relative to normalized total tau levels and compared to *Scn1a* +/+ (129) and *Scn1a* +/Δ (129) mice. Similarly, individual *Scn1a* Δ/Δ (129) samples showed increased phosphorylated p38 MAPK levels (pp38 MAPK). Total tau levels and its phosphorylation at sites tauS202, tauS214, tauS422, and tauPHF1 (S396 + S404) as well as total p38γ levels were comparable between *Scn1a* +/+, *Scn1a* +/Δ, and *Scn1a* Δ/Δ (129) mice [*n* = 4 to 5 mice per genotype; ****P ≤ 0.0001; ns (ANOVA)]. (**B**) Schematic of the experimental design for p38γ therapeutic study before pilocarpine-mediated seizure induction. (**C**) Seizure severity and latency (top), survival (bottom left), and mean seizure severity (bottom right) after pilocarpine induction in 6-week-old *tau*T205T/T and *tau*T205A/A mice, following prior AAV treatment at 4 weeks of age. Wild-type B6 (*tau*T205T/T) mice pretreated with p38γ^CA^ (*tau*T205T/T.p38γ^CA^) show significantly prolonged latency to develop more severe seizures (**P = 0.0049), improved survival (**P = 0.0054), and reduced mean seizure severity (**P = 0.0015) as compared to *tau*T205T/T.control mice. In contrast, *tau*T205A/A.p38γ^CA^ mice did not display significant differences in seizure latency, mean seizure severity, or survival compared to both *tau*T205T/T.control and *tau*T205A/A.control groups. Accordingly, *tau*T205A/A.p38γ^CA^ showed significantly reduced latency (***P = 0.0007), increased mean seizure severity (***P = 0.0003), and increased mortality (***P = 0.0006) compared to *tau*T205T/T.p38γ^CA^ mice. *n* = 15 to 20 mice per group (ANOVA and Mantel-Cox test, respectively).

We hypothesized that p38γ exerts its protection during epileptic events through phosphorylation of tau at T205, similar to what we have previously shown for AD (*9*). Therefore, cortical tissue was extracted from *Scn1a* (129) mice of all genotypes at P14, at which point, homozygous *Scn1a* Δ/Δ (129) mice exhibited seizures preceding death. Tau levels were indistinguishable between *Scn1a* +/+ (129) (=wild-type controls), *Scn1a* +/Δ (129), and *Scn1a* Δ/Δ (129) mice (Fig. 2A). However, *Scn1a* Δ/Δ (129) cortices were presented with significantly increased tau phosphorylation at T205 (ptauT205) (Fig. 2A). This was further confirmed by significantly increased ptauAT8 (ptauS202/T205) signals in the absence of altered ptauS202 levels, indicating targeted phosphorylation at tauT205 during seizures. While total p38γ levels were incomparable across the genotypes, individual *Scn1a* Δ/Δ (129) mice showed markedly increased signals for active, phosphorylated p38 MAPK, suggestive of engaged activation pathways at the time of sample collection. Short-term transient activation of MAPKs during seizure events has been reported before (*18*). No overt changes to tau phosphorylation at other probed sites [S202, S214, S396/S404 (=PHF1), and S422] were detected in *Scn1a* Δ/Δ (129) mice before death. However, we found pronounced phosphorylation of tau at S262, which is not a neuronal p38γ target site (*9*) but one of two sites phosphorylated by the kinase microtubule affinity-regulating kinase 2 (MARK2) (*19*). Thus, general up-regulation of tau phosphorylation does not account for elevated phosphorylation at distinct sites, suggesting that phosphorylation at these occurs in response to acute neuronal hyperexcitation during seizures, in an attempt to alleviate excitotoxic induction as an endogenous protective mechanism in *Scn1a* Δ/Δ (129) mice. In addition, increased tau pT205 was observed in *Scn1a* +/Δ.p38γ^CA^ mice (fig. S2D), further crediting T205 in tau as a primary site of p38γ in mediating therapeutic benefits in DS.

Only a small proportion of epilepsies have a known genetic cause (*20*). Therefore, to probe the protective mechanism of p38γ in further detail in seizures with no genetic cause, we adopted a chemically induced seizure model to study both acute and chronic aspects of seizures. To assess the preventative effects of p38γ on acute seizure induction, wild-type B6 mice (referred to as *tau*T205T/T) were treated by intravenous delivery of neurotropic AAV for neuronal expression of p38γ^CA^ (*tau*T205T/T.p38γ^CA^) or a control (*tau*T205T/T.control), 2 weeks before administration of pilocarpine (Fig. 2B). Pilocarpine, a muscarinic receptor agonist, elicits sustained neuronal hyperexcitation and induction of excitotoxic mechanisms, in turn, triggering status epilepticus (SE) in rodents (*21*). Pilocarpine-treated *tau*T205T/T.control mice developed severe seizures and frequently did not survive SE (Fig. 2C). In contrast, *tau*T205T/T.p38γ^CA^ mice experienced significantly longer latency to seizure onset, reduced seizure severity, and seizure-induced mortality (Fig. 2C). To probe the mechanism by which p38γ facilitates seizure amelioration, the genetically modified B6 mouse strain, *tau*T205A/A, was used; this strain harbors a mutated murine *Mapt* locus (the gene encoding tau protein) at the residue corresponding to human T205, encoding phosphorylation-defective *tau*T205A/A (Fig. 2B). When we administered pilocarpine, the protective effects of p38γ were lost in *tau*T205A/A mice, as seizure severity, latency, and mortality did not differ from those of control-treated *tau*T205T/T mice, irrespective of pretreatment with p38γ^CA^, and trends toward acceleration of seizure onset and mortality appeared in *tau*T205A/A mice from both treatment groups (Fig. 2C).

### p38γ treatment after seizure induction is protective

**Fig. 3.**
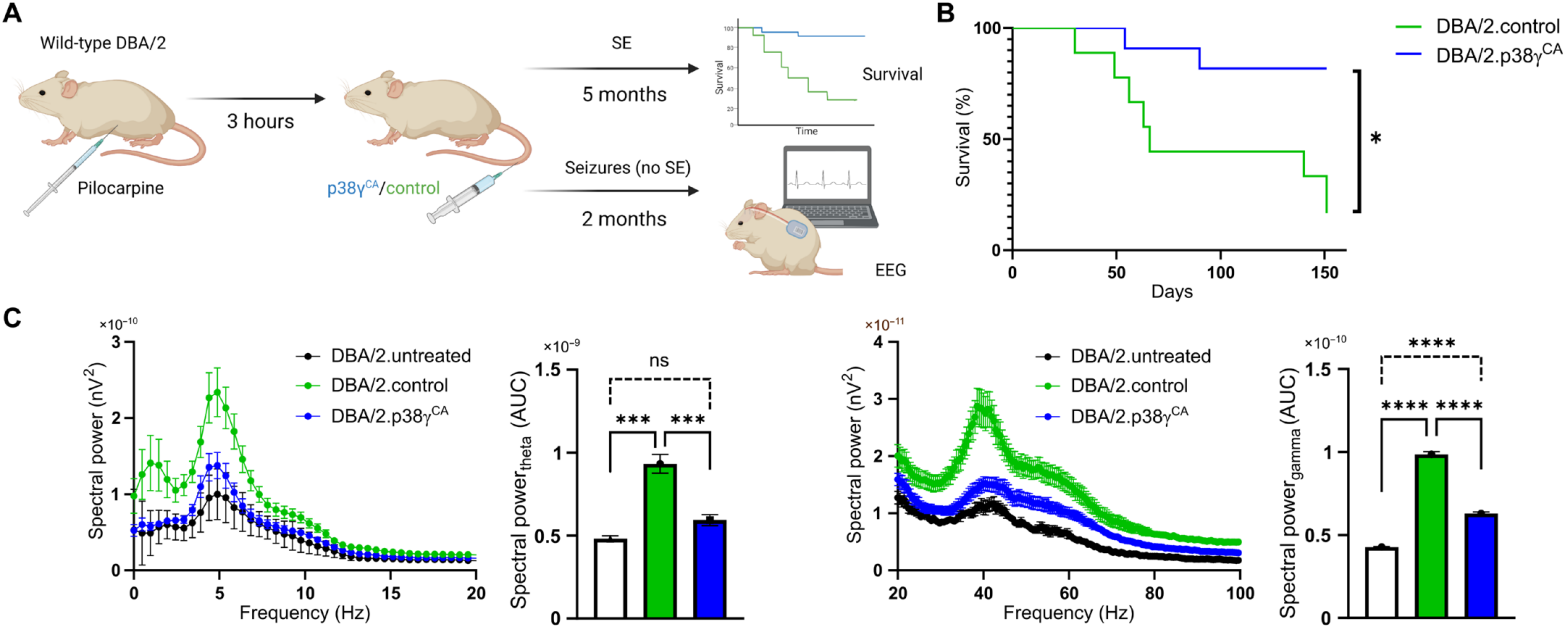
p38γ^CA^ treatment after seizure induction is protective. (**A**) Schematic of experimental strategy for p38γ therapeutic study following pilocarpine-mediated seizure induction. (**B**) Survival curve following pilocarpine induction shows improved DBA/2.p38γ^CA^ survival compared to DBA/2.control mice during a 4-month recording period following pilocarpine injection (day 0). *n* = 9 to 11 mice per group; *P = 0.0150 (Mantel-Cox test). (**C**) EEG power spectral density profiles during wake in DBA/2 mice 2 months following pilocarpine injection. DBA/2.control mice exhibited significantly increased high and low spectral power compared to untreated DBA/2 mice (theta: ***P = 0.0002; gamma: ****P ≤ 0.0001). DBA/2.p38γ^CA^ mice showed significantly reduced high and low spectral power, compared to DBA/2.control mice (theta: ***P = 0.0010; gamma: ****P ≤ 0.0001). DBA/2.p38γ^CA^ theta range was not significantly (ns) different from that of DBA/2.untreated mice but remained moderately increased in the gamma range (****P ≤ 0.0001). *N* = 4 mice per group (ANOVA).

Temporal lobe epilepsy (TLE) emerges as the most common focal epilepsy; patients experience recurrent seizures (which are unremitting without invasive temporal lobe resection), accompanied by brain-wide pathological changes on a cellular and structural level (*22*), cognitive decline, and high risk of seizure-related mortality. Therefore, to assess its therapeutic potential after seizure onset, p38γ was administered only following pilocarpine induction. Here, we used DBA/2 mice that exhibit seizures upon pilocarpine injection but in the absence of high mortality rates, and DBA/2 mice that reach SE later develop chronic spontaneous recurrent seizures (SRS) after recovery from pilocarpine, thus recapitulating TLE (*23*). After pilocarpine administration, DBA/2 mice that exhibited one or more tonic-clonic seizures (see Materials and Methods) were administered an intravenous dose of neurotropic AAV for neuronal expression of p38γ^CA^ (DBA/2.p38γ^CA^) or a control (DBA/2.control; Fig. 3A). Daily observation showed that all mice that developed SE exhibited symptoms consistent with SRS, including partial and generalized seizures from 48 hours after injection, irrespective of AAV treatment. Consistent with previous reports (*23*), DBA/2.control mice experienced mortality during the chronic SRS phase (48 hours to 5 months after treatment). In contrast, significantly reduced incidence of SRS-induced mortality was observed in treated DBA/2.p38γ^CA^ (Fig. 3B). Given the incidence of chronic seizures and high susceptibility to SRS-induced mortality in DBA/2 mice, separately, mice that experienced one or more seizures but did not develop SE upon pilocarpine administration (i.e., a milder seizure model) were used to assess long-term neuronal network alterations. EEG electrodes were implanted 2 months after treatment and compared to an untreated group that received neither pilocarpine nor AAVs (DBA/2.untreated). EEG recordings revealed a nonsignificant trend toward increased epileptiform discharges following pilocarpine, irrespective of p38γ^CA^ or control transgene delivery (fig. S2E). DBA/2.control mice demonstrated significantly increased high and low spectral power (Fig. 3C) compared to DBA/2.untreated mice. Conversely, DBA/2.p38γ^CA^ mice displayed normalized spectral power.

## DISCUSSION

### p38γ kinase treatment protects DS mice

Here, we demonstrate the protective effects of p38γ on several, clinically relevant aspects of DS. Seizure-induced mortality was significantly reduced in *Scn1a* +/Δ (B6) mice. Thus, we suggest that enhancing p38γ kinase activity holds promise for attenuating the severity and/or frequency of seizure episodes responsible for SUDEP in patients with DS. Moreover, aberrant hippocampal EEG profiles were normalized in *Scn1a* +/Δ.p38γ^CA^ mice on both genetic background strains, demonstrating a role for p38γ in reducing hyperexcitation-induced neuronal network aberrations in the hippocampus. Aberrant behaviors were reduced in *Scn1a* +/Δ.p38γ^CA^ (129) mice, while the high mortality of *Scn1a* +/Δ.control (B6) mice rendered conclusive behavioral testing in comparison to the surviving *Scn1a* +/Δ.p38γ^CA^ (B6) impossible. p38γ^CA^ expression in controls of both backgrounds led to disrupted EEG profiles but without overt behavioral changes. Therefore, the therapeutic benefits of increasing p38γ activity may be limited to individuals with epilepsy. Together, our results provide evidence that p38γ acts through interrupting cellular mechanisms that trigger SUDEP in DS by preventing the imbalance of neuronal circuits and that this network preservation may ameliorate cellular processes, leading to downstream cognitive and behavioral symptoms in DS.

### p38γ treatment after seizures is protective

We demonstrated that neurotropic p38γ kinase treatment, following seizure onset, imparted significant protection from SRS-induced mortality in mice. Considering that relevant therapeutic gene expression occurs only 1 to 2 weeks after AAV administration, our data suggest that p38γ activity might alleviate seizure-induced mortality in patients with chronically recurring seizure disorders such as TLE. Furthermore, following a seizure event, we unveil the presence of persistent network abnormalities, while p38γ activity disengages epileptiform discharges from downstream toxic events and restores aberrant neuronal network functionality after epileptic events.

### tauT205 is a protective site in epilepsy

We previously reported that tauT205 is a primary site for p38γ-mediated neuroprotection in AD (*9*, *10*). Here, we show the significance of tau phosphorylation at T205 in both genetic and chemically induced seizure models. The significant up-regulation of ptauT205 in *Scn1a* Δ/Δ (129) mice during overt seizures suggests that tauT205 hyperphosphorylation is an early event during neuronal hyperexcitation. Similarly, p38γ^CA^ in *Scn1a* +/Δ (B6), but not *Scn1a* +/+ (B6), mice underwent increased activation by phosphorylation. Together, this implies an engagement of an endogenous protective pathway during seizures involving p38γ and ptauT205. However, this alone is not sufficient to rescue the complete loss of function of Scn1a, as homozygosity in mice and humans alike is lethal (*15*, *24*). Mechanistically, we have previously shown that phosphorylation of tau at T205 by p38γ disengages postsynaptic signaling complexes that govern excitotoxicity in AD (*6*, *9*). Accordingly, we speculate that increased p38γ activity limits excitotoxicity in epilepsy and therefore SE-induced deaths via T205 phosphorylation of tau and the resulting inhibition of postsynaptic excitotoxic signaling. The importance of the T205 site in exerting p38γ-mediated protection in the context of epilepsy was further underlined by virtually completely diminished antiepileptic effects of p38γ^CA^ pretreatment in the absence of tauT205 in mice (=*tau*T205A/A). Nevertheless, we cannot formally rule out that other, unknown molecular targets of p38γ may contribute to these protective effects. Together, p38γ effectively ameliorates acute epileptic mechanisms that trigger SE and SE-induced death, and phosphorylation of tau at T205 is imperative for this protection. Lack of function at this site (=T205A/A) renders p38γ incapable of tau phosphorylation, in turn, mitigating its therapeutic effects and possibly accelerating epileptogenic processes.

The absence of tau hyperphosphorylation in *Scn1a* Δ/Δ (129) mice argues against promiscuous kinase activity during epileptic events, but rather specific activation of kinases, such as p38γ. *Scn1a* Δ/Δ (129) mice were presented with significant tau phosphorylation at S262, which is not a p38γ target site (*9*). S262 phosphorylation is mediated by MARK2 (*19*). Phosphorylation of tau at S262 compromises its interaction with microtubules (*19*, *25*), raising the interesting idea that microtubule dynamics are changed during seizure events. However, the role of MARK2 in epilepsy remains largely unexplored, and further investigation is required.

In summary, we show that, before seizure onset, overactivation of p38γ significantly attenuates aberrant neuronal function, disrupted behaviors, and seizure-induced mortality in both epilepsy mouse models. p38γ appears to disengage tau from its epilepsy-associated pathway at the postsynapse, alleviating downstream clinical outcomes. This is mediated through phosphorylation at tauT205. Furthermore, we demonstrate that p38γ administration following seizure onset significantly restores neuronal network patterns in a pilocarpine model of epilepsy and reduces molecular processes, leading to seizure-induced mortality. Thus, we propose increasing p38γ activity as a multiuse therapeutic target both in preventing and in treating diseases characterized by network dysfunction and excitotoxicity.

## MATERIALS AND METHODS

### Mice

129S6/SvEvTacAusb (129) mice were obtained from Australian BioResources. C57Bl/6J (B6) and DBA/2J (DBA/2) mice were obtained from the Animal Resources Centre, Australia. All animals were housed and tested in the Central Animal Facility (CAF) at Macquarie University. Mice were maintained with free access to food and water on 12-hour light/12-hour dark cycles in individually ventilated cages. All experiments involving animals were approved by the Macquarie University Animal Ethics Committee and carried out in accordance with Australian legislation (AEC #2017/053, #2017/033, and #2018/019). All testing and analyses were done by researchers blinded to genotypes and treatments. Mice were randomly selected. Mice within CAF were routinely screened for pathogens and cleared for experimental use. Experimental mice were weighed twice weekly during the period of the study, and mice with nonrelated health issues (e.g., malocclusion and hydrocephaly) were excluded from the study. Animal numbers used in experiments are outlined in table S1.

### DS mouse model

The first exon of the murine *Scn1a* gene (Ensembl, ENSMUSG00000064329) was targeted to remove the endogenous start codon (fig. S1A) by CRISPR-mediated gene targeting using two guides (guide 1: 5′-TTTAATGTTCTCCACGTTTC-3′; guide 2: 5′-TGCGCCTTTCAATAGCTGCA-3′). These single-guide RNAs were rationally designed using a computational tool to minimize off targets (https://bioinfogp.cnb.csic.es/tools/breakingcas/) and were produced using a noncloning method whereby a T7-conjugated forward primer generates a linear template by polymerase chain reaction, as previously described (*26*). The guides were incubated with S.p.Cas9 protein (New England Biolabs, #M0646T) to form ribonucleoprotein (RNP) complexes. RNPs were electroporated (NEPA21, Nepagene) into fertilized 129 zygotes with the respective concentrations: 320 ng/μl of Cas9 and 80 ng/μl of each guide. The selected founder displayed a deletion at the endogenous *Scn1a* locus. Sanger sequencing (Macrogen, South Korea) of the edited allele (fig. S1A) revealed a 138–base pair deletion that removed the critical start codon. This founder was bred to establish the colony, and mice were then either maintained on a 129 background [referred to as *Scn1a* +/Δ (129)] or crossed once with B6 mice to generate an F_1_ hybrid strain [referred to as *Scn1a* +/Δ (B6)].

### tauT205A mouse model

Single–base pair point mutation of T194 codon in murine *Mapt* (analogous site to human *tau*T205) was previously generated using the CRISPR-Cas9 gene editing technology in B6 zygotes as described (*10*), altering the encoding amino acid from threonine to alanine, rendering tau phosphorylation deficient at 194. Correctly targeted mice were bred to homozygosity (*tau*T205A/A) and maintained homozygous for use in this study.

### AAV treatment

AAV constructs were generated and packaged as previously described (*10*). Briefly, the transgenes, CA p38γ, p38γ^CA^ (*p38*γAsp179Ala), and a nontherapeutic control encoding the green fluorescent protein (GFP), under the control of the neuron-specific promoter h-synapsin, were packaged, respectively, into the replication-deficient neurotropic AAV, AAV-PHP.B (*27*): AAV-PHP.B-syn1-*p38γ^CA^* and AAV-PHP.B-syn1-*eGFP*. For treatment in DS mice, 40 μl of AAV particles (5 × 10^13^ viral particles/ml) was administered by temporal vein injection into cryo-anesthetized P0 *Scn1a* mice (*28*). For treatment in pilocarpine-induced seizure mice, 100 μl of AAV particles was administered by tail vein injection. Wild-type B6 and *tau*T205A/A (B6) mice were treated at 4 weeks of age (5 × 10^12^ viral particles/ml), 10 days before seizure induction. DBA/2 mice were treated at 10 weeks of age (5 × 10^13^ viral particles/ml), 3 hours after pilocarpine administration. Treatment of *Scn1a* mice was conducted by AAV administration in at least three litters per AAV treatment group. Treatment of *tau*T205T/T, *tau*T205A/A, and DBA/2 mice was performed in duplicate (equal numbers of mice from each cohort were assigned to each AAV treatment group).

### Seizures

Pilocarpine-mediated seizure induction was performed as previously described (*29*). Briefly, *n*-methyl scopolamine (1 mg/kg) was administered intraperitoneally (ip), to prevent peripheral effects, 30 min before a bolus dose of pilocarpine (375 mg/kg, ip) in 6-week-old male B6 (referred to as *tau*T205T/T) or *tau*T205A/A mice and of pilocarpine (300 mg/kg, ip) into male DBA/2 mice. Seizures were scored using a modified Racine scale: 0, no seizures; 1, immobility; 2, tail extension; 3, mild tremors (“wet-dog shakes”); 4, partial clonus; 5, tonic-clonic seizure (<1-min duration); 6, tonic-clonic seizure with loss of consciousness (>5-min duration); and 7, SE [tonic-clonic seizure with loss of consciousness (>20-min duration) or seizure-induced death]. Mice with labored respiration or respiration arrest were immediately euthanized in accordance with ethics protocols. After 3 hours, surviving mice were administered with diazepam (10 mg/kg) to alleviate convulsive seizures. DBA/2 mice that exhibited one or more grade 5 seizures (but did not reach SE) were kept for EEG recording 2 months after pilocarpine administration. DBA/2 mice developing SE were monitored for rates of SRS-induced mortality for 5 months following pilocarpine administration. Mice that died within 48 hours following pilocarpine administration were excluded from the survival study. Mice of each treatment group and strain/genotype were used from at least two separate experiments.

### EEG recordings

EEG electrodes were implanted into the hippocampus of 6-month-old DS mice of mixed sexes and 18-week-old pilocarpine-treated male DBA/2 mice as previously described (*16*). Briefly, under anesthesia, mice were mounted onto a stereotaxic frame, and electrodes were surgically implanted intracranially into the right hippocampus. After a minimum of 5 days after surgery, individually housed mice were placed on EEG receiver platforms (DSI) in their home cages, and the Ponemah software (DSI) was used to record EEG data during a 48-hour time period of free movement. Analysis of recordings was completed using NeuroScore software (DSI).

### Behavior testing

Novelty-induced activity was assessed in mice using the open-field test paradigm as previously described (*9*, *30*). Briefly, the open field consists of 40-cm by 40-cm, dimly lit, sound-insulated enclosures. Sex-mixed groups of animals were individually placed in the field center and recorded using an aerial video recording camera and Empia Capture software for 10 min. Behavioral analysis was completed using ANY-maze video tracking software (Stölting). Cohorts of mice were tested at either 3 to 4 weeks of age or 2 months of age and at 5 to 6 months of age.

### Western blotting

Following transcardial perfusion with 1× phosphate-buffered saline (PBS) (pH 7.4), mouse cortices were extracted and homogenized at 10 μg/μl in cold radioimmunoprecipitation assay (RIPA) buffer [20 mM Mops (pH 7), 1% Triton X-100, 0.25%, Na-deoxycholate, 0.1% SDS, 150 mM NaCl, 2 mM EGTA (pH 8), 5 mM EDTA (pH 8), 30 mM NaF, 60 mM β-glycerophosphate, 20 mM Na-pyrophosphate, 1 mM Na-orthovanadate, 5 μM pepstatin A, 1 μM dithiothreitol, and protease inhibitor (one tablet per 10 ml)] using a Dounce homogenizer (Heidolph). Samples were then briefly sonicated and centrifuged at 14,000 rpm for 30 min at 4°C to generate a RIPA-soluble fraction. Protein concentrations were determined by bicinchoninic acid assay (BCA assay) (Pierce, Thermo Fisher Scientific). Western blotting was performed as previously described (*9*, *10*). Antibodies used were as follows: anti-Nav1.1 (Neuromab, K74/71), glyceraldehyde-3-phosphate dehydrogenase (anti-GAPDH; Millipore, MAB374), anti-hemagglutinin (HA; Cell Signaling Technology, clone C29F4: 3724S), anti-GFP (Abcam, ab290), anti-Tau5 (Invitrogen, AHB0042), anti–phosphorylated T205 tau (Abcam, ab181206), anti–phosphorylated S214 tau (Abcam, ab170892), anti–phosphorylated S422 tau (Abcam, ab79415), anti–phosphorylated S202 tau (Abcam, ab108387), anti–phosphorylated S262 tau (Invitrogen, 44-750G), anti–phosphorylated tau AT8 (Ser^202^/Thr^205^) (Invitrogen, MN1020), anti–phosphorylated tau PHF1 (Ser^396^/Ser^404^) (Dementia Research Centre in-house production), anti-p38γ (R&D Systems, AF1347), anti–phosphorylated p38 (Cell Signaling Technology, 4631), and secondary antibodies coupled to horseradish peroxidase. Western blotting for each antibody was conducted in duplicate with at least three biological replicates per genotype and treatment group. Membranes were imaged by chemiluminescence using a ChemiDoc system (Bio-Rad). Densitometric analysis of immunoblots was performed using Image Lab 6.0 software.

### Immunohistochemistry

Following transcardial perfusion in 1× PBS, left brain hemispheres were fixed in 4% paraformaldehyde overnight (4°C) and processed using an Excelsior tissue processor (Thermo Fisher Scientific). Samples were then embedded in paraffin and sectioned at a thickness of 3 μm (Thermo Fisher Scientific, HM325). Immunostaining was performed as previously described (*10*). Briefly, following antigen retrieval, histological sections were blocked and permeabilized (5% donkey serum + 0.1% Triton X-100). Primary antibodies, anti-GFP (Abcam, ab290) and anti-HA (Cell Signaling Technology, clone C29F4: 3724S), to visualize AAV-transgene expression, were incubated on slides overnight (4°C). Alexa fluorophore-coupled secondary antibodies 488 and 568 (Molecular Probes) diluted in blocking buffer (5% donkey serum) were incubated on slides at room temperature for 1 hour before 4′,6-diamidino-2-phenylindole staining for 10 min. Slides were mounted for confocal imaging using an AxioScan microscope slide scanner (Zeiss).

### Statistical analyses

For analysis of a single variable, unpaired *t* tests were used. For multiple data groups, one-way analysis of variance (ANOVA), with Tukey’s multiple comparisons test, was used. For survival analysis, log-rank (Mantel-Cox) test was performed. Power spectral densities from hippocampal EEG recordings during periods of wake were determined by area under the curve analysis, followed by unpaired *t* tests or one-way ANOVA. Spike scoring was performed by taking the number of spikes detected from a 24-hour recording period and calculated as the number of spikes per hour. All data are presented as ±SEM.

### Diagrams

Diagrams for experimental workflows were created using BioRender.com.

## Notes

View/request a protocol for this paper from *Bio-protocol*.
