# Supplementary material for: Treatment of epilepsy using a targeted p38γ kinase gene therapy
Source: Sci Adv. 2022 Dec 2;8(48):eadd2577. doi: 10.1126/sciadv.add2577 (PMC10936047; doi:10.1126/sciadv.add2577)
Supplement: Supplementary file 1 — Figs. S1 and S2 Table S1 [file sciadv.add2577_sm.pdf]

Science Advances
AAAS

Supplementary Materials for

Treatment of epilepsy using a targeted p38γ kinase gene therapy

Nicolle Morey *et al.*

Corresponding author: Lars M. Ittner, lars.ittner@mq.edu.au

Sci. Adv. **8**, eadd2577 (2022)
DOI: 10.1126/sciadv.add2577

This PDF file includes:

Figs. S1 and S2
Table S1

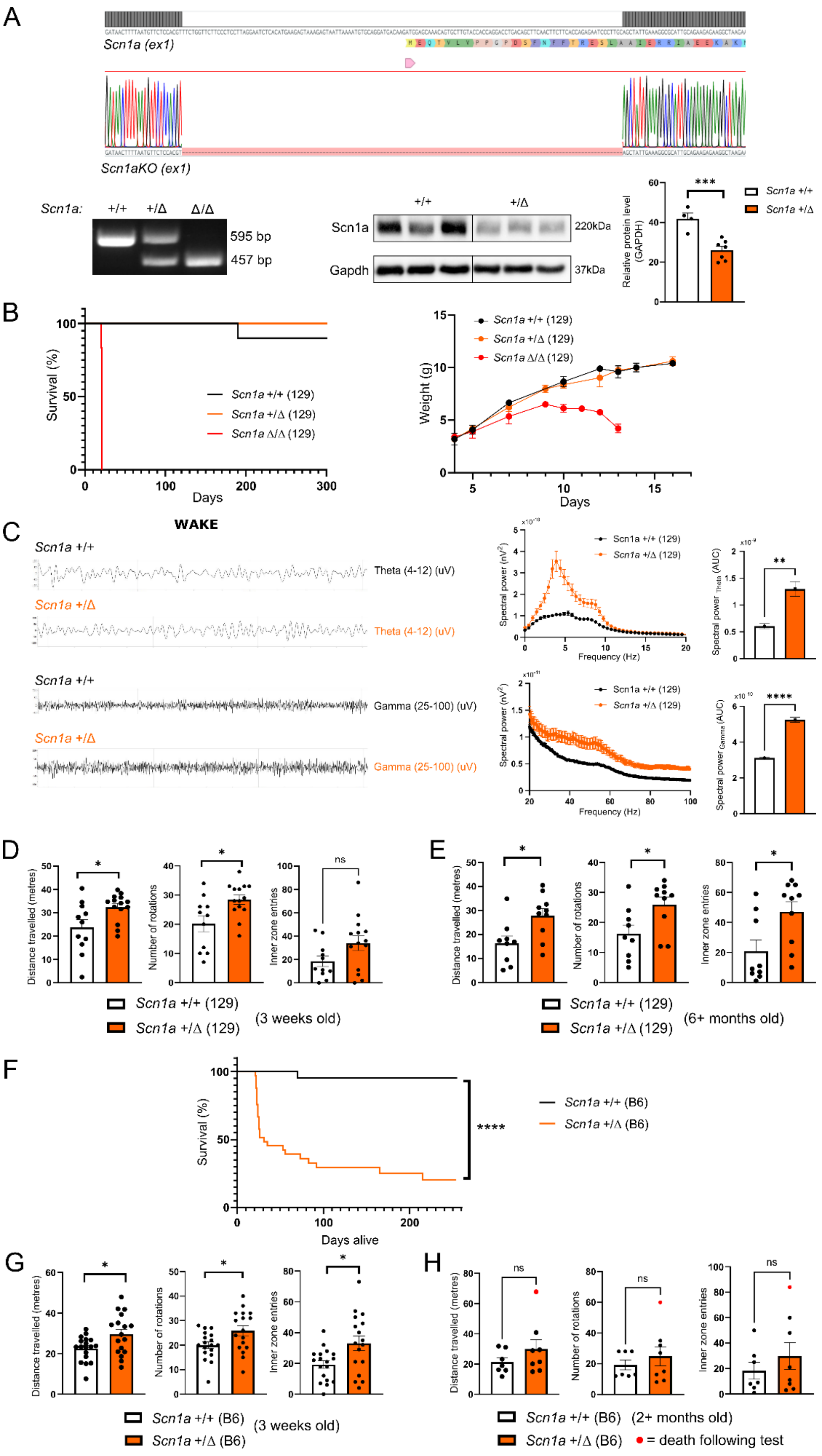
A
Scn1a (ex1)
Scn1aKO (ex1)
Scn1a: +/+ +/Δ Δ/Δ
595 bp
457 bp
+/+ +/Δ
Scn1a
Gapdh
220kDa
37kDa
Relative protein level (GAPDH)
***
Scn1a +/+
Scn1a +/Δ
B
Survival (%)
Days
Scn1a +/+ (129)
Scn1a +/Δ (129)
Scn1a Δ/Δ (129)
Weight (g)
Days
C
WAKE
Scn1a +/+
Theta (4-12) (uV)
Scn1a +/Δ
Theta (4-12) (uV)
Scn1a +/+
Gamma (25-100) (uV)
Scn1a +/Δ
Gamma (25-100) (uV)
Spectral power (nV²)
Frequency (Hz)
Spectral power Theta (AUC)
**
Spectral power Gamma (AUC)
****
D
Distance travelled (metres)
Number of rotations
Inner zone entries
*
*
ns
Scn1a +/+ (129)
Scn1a +/Δ (129)
(3 weeks old)
E
*
*
*
Scn1a +/+ (129)
Scn1a +/Δ (129)
(6+ months old)
F
Survival (%)
Days alive
****
Scn1a +/+ (B6)
Scn1a +/Δ (B6)
G
*
*
*
Scn1a +/+ (B6)
Scn1a +/Δ (B6)
(3 weeks old)
H
ns
ns
ns
Scn1a +/+ (B6)
(2+ months old)
Scn1a +/Δ (B6)
● = death following test

Fig. S1.

Depletion of *Scn1a* in Dravet syndrome (DS) mice. (A) Validation of *Scn1a* targeting in a *Scn1a* knockout (KO) mouse model on the 129 background strain. Top: Next Generation Sequencing (NGS) alignment of *Scn1a* wild-type gene sequence of exon 1 (above), flanking the initiation sequence, ATG, compared to *Scn1a*KO exon1 gene sequence (below), extracted from founder DNA (bottom). Bottom left: Genotyping of *Scn1a*KO by PCR demonstrates a wild-type band at 595bp and a mutant band at 457bp, reflecting the 138bp deletion in exon1 of *Scn1a*. Bottom right: western blotting verified significant loss of Scn1a expression in the brain of *Scn1a* +/Δ mice (n=7) compared to *Scn1a* +/+ littermates at 21 days post-natal (n=4; ***, p=0.0010 [Student t-test]). Gapdh loading control was run in duplicate on a separate blot due to the large size of Scn1a protein. **(B)** Survival curve (left) of homozygous (Δ/Δ), heterozygous (+/Δ) and wild-type (+/+) *Scn1a* mice on the 129 background over a 300-day recording period. *Scn1a* Δ/Δ (129) had significantly higher mortality than wild-types and heterozygotes while survival of *Scn1a* +/Δ (129) and *Scn1a* +/+ (129) was not significantly altered (n=≥31 per group; ****, p<0.0001; ns, not significant; [Mantel-Wilcox test]). Body weights (right) during pre-weaning period P5 – P17, were significantly reduced by P13 in *Scn1a* Δ/Δ (129) mice compared to *Scn1a* +/+ and *Scn1a* +/Δ littermates (n=4-7; ****, p<0.0001 [Student t-test]). **(C)** Hippocampal EEG profiles of *Scn1a* (129) mice at 6 months of age during periods of wake. Left: example trace recordings of theta (4-12Hz) and gamma (25-100Hz) frequencies, demonstrating no epileptiform discharges. Right: *Scn1a* +/Δ mice show significantly increased theta and gamma spectral power (n=4-5; **, p=0.0012 (theta); ****, p=<0.0001 (gamma) [Student t-test]). **(D-E)** Behavioural analysis in *Scn1a* +/+ and *Scn1a* +/Δ (129) mice tested in the open field task. **(D)** At 3-4 weeks of age mice *Scn1a* +/Δ (129) mice travelled significantly further distances, exhibited more circling and entered the inner zone of the field more often than wild-type littermates (*, p=0.0191 (distance); *, p=0.0110 (circling); ns, not significant (zone) [Student t-test]). N=11-14 per genotype. **(E)** At 6-9 months of age mice *Scn1a* +/Δ (129) mice travelled significantly further distances, exhibited more circling and entered the inner zone of the field more often ((*, p=0.0139 (distance); *, p=0.0217 (circling); *, p=0.0172 (zone) [Student t-test]) than wild-type littermates. N=9-10 per genotype. **(F)** Survival of untreated *Scn1a* +/Δ (B6) mice (n=33) was significantly worse than wild-type littermates (n=24) over a 260-day recording period (****, p=<0.0001 [Mantel-Cox]). **(G-H)** Behavioural analysis in untreated *Scn1a* +/+ and *Scn1a* +/Δ (B6) mice tested in the open field task. **(G)** At 3-4 weeks of age *Scn1a* +/Δ (B6) mice travelled significantly further distances, exhibited more circling entered the inner zone of the field more often than wild-type littermates (n=17-18 per genotype; (*, p=0.0154 (distance); (*, p=0.0197 (circling); (*, p=0.0116 (zone) [Student t-test]). At >2 months of age mice *Scn1a* +/Δ (B6) behaviours were not significantly different to wild-type littermates (ns, not significant [Student t-test]) due to mortality, and mice that exhibited most abnormal behaviours died from seizures within 2 weeks following testing (red symbols).

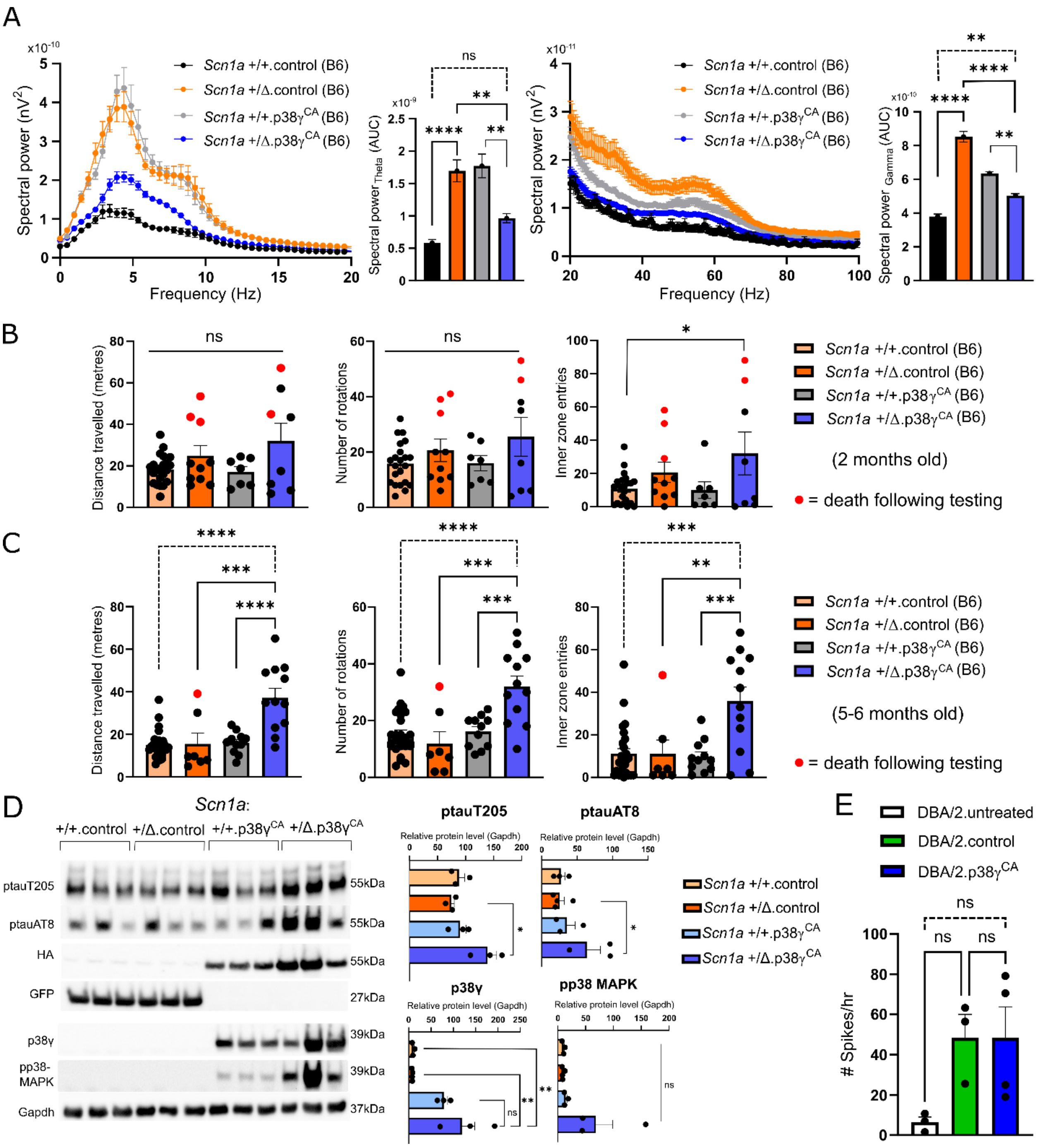

Fig. S2.

Extended EEG, behavioural analysis and western blotting of p38γ^{CA}-treated *Scn1a* (B6) and pilocarpine-injected DBA/2 mice (A) EEG power spectral density profiles during wake phases in treated *Scn1a* (B6) mice at 5-6 months of age. *Scn1a* +/Δ.control mice demonstrated significantly elevated power spectral densities for theta and gamma frequencies when compared to *Scn1a* +/+.control mice, (****, p=<0.0001). When compared to *Scn1a* +/Δ.control mice, *Scn1a* +/Δ.p38γ^{CA} mice demonstrated significantly reduced spectral power in both theta and gamma frequencies (**, p=0.022 (theta); ****, p<0.0001 (gamma). *Scn1a* +/Δ.p38γ^{CA} mice

exhibited theta power not significantly different to *Scn1a* +/+.control mice, but gamma power remained significantly different to *Scn1a* +/+.control mice (**, p=0.0012; ns, not significant). *Scn1a* +/+.p38γ^{CA} mice exhibited consistently elevated theta and gamma power compared to *Scn1a* +/+.control and *Scn1a* +/Δ.p38γ^{CA} mice (n=4-6 per group; **, p=≤0.0014 [ANOVA]). **(B-C)** Open field behavioural analysis of treated *Scn1a* (B6) mice. **(B)** At 2 months of age, mice from *Scn1a* mice of all treatment groups did not significantly differ behaviourally in distance travelled (ns, p=0.1407), circling (ns, p=0.1934) or inner zone field entries (ns, p=0.1258) with the exception of *Scn1a* +/Δ.p38γ^{CA} mice, who entered significantly more than *Scn1a* +/+.control mice (*, p=0.0450). N=7-22 per group. **(C)** At 5-6 months of age, *Scn1a* +/Δ.p38γ^{CA} mice travelled significantly further distances (***, p=0.0001; ****, p=<0.0001), exhibited more circling behaviour (***, p=0.0006; ****, p=<0.0001) and entered the inner zone more than all other treatment groups (**, p=0.0056; ***, p=0.0007). Mice that exhibited most abnormal behaviours died from seizures within 2 weeks following testing (red symbols). N=7-12 per group. **(D)** Western blot of ptauT205, ptauAT8 (S202+T205), p38γ and phoshorylated p38 MAPK (pp38 MAPK) levels in treated *Scn1a* (B6) mice. Significantly increased ptauT205 and ptauAT8 levels were observed in *Scn1a* +/Δ.p38γ^{CA} mice compared to *Scn1a* +/+.control mice, and trends towards increased ptauT205 levels compared to *Scn1a* +/+. p38γ^{CA} and *Scn1a* +/Δ.control groups, though not to statistical significance (*, p=0.0159 (T205); *, p=0.0369 (AT8); ns, not significant [ANOVA]). Significantly increased p38γ levels were observed in *Scn1a* +/Δ.p38γ^{CA} treated mice compared to control groups of both genotypes and was not significantly different to *Scn1a* +/+. p38γ^{CA} mice. *Scn1a* +/+. p38γ^{CA} mice p38γ levels were not significantly altered from control levels (** p=≤0.0060; ns, not significant [ANOVA]). P38 MAPK phosphorylation levels were not significantly altered following p38γ^{CA} administration, though a trend towards increased phosphorylation in *Scn1a*.p38γ^{CA} mice was observed, most prominently in *Scn1a* +/Δ.p38γ^{CA}, and this increase corresponded with those mice demonstrating higher p38γ levels (n=3-4 per group; ns, not significant [ANOVA]). **(E)** Spike scoring from EEG analysis of DBA/2 mice 2 months following pilocarpine administration and AAV treatment. Number of spikes per hour showed a non-significant trend towards increase in the pilocarpine treated groups, and irrespective of p38γ^{CA} or control AAV treatment following pilocarpine, spike number was not significantly altered (n=4 per group; ns, not significant [ANOVA]).

Experiment	Mouse strain	Sex	
		**M**	**F**
Survival	*Scn1a* +/+ (129)	28	20
	Scn1a +/Δ (129)	53	39
	Scn1a Δ/Δ (129)	22	9
	Scn1a +/+ (B6)	17	11
	Scn1a +/Δ (B6)	24	17
	Scn1a +/+.p38γ^{CA} (B6)	6	5
	Scn1a +/Δ.p38γ^{CA} (B6)	11	11
	Scn1a +/+.control (B6)	15	14
	Scn1a +/Δ.control (B6)	20	18
	DBA/2.p38γ^{CA}	11	n/a
	DBA/2.control	9	n/a
	*tau*T205T/T.p38γ^{CA}	20	n/a
	*tau*T205T/T.control	20	n/a
	*tau*T205A/A.p38γ^{CA}	15	n/a
	*tau*T205A/A.control	16	n/a
EEG	*Scn1a* +/+ (129)	2	3
	Scn1a +/Δ (129)	4	0
	Scn1a +/+.p38γ^{CA} (129)	1	3
	Scn1a +/Δ.p38γ^{CA} (129)	5	0
	Scn1a +/+.control (129)	3	1
	Scn1a +/Δ.control (129)	2	1
	Scn1a +/+.p38γ^{CA} (B6)	2	2
	Scn1a +/Δ.p38γ^{CA} (B6)	3	3
	Scn1a +/+.control (B6)	3	1
	Scn1a +/Δ.control (B6)	3	1
	DBA/2.p38γ^{CA}	4	n/a
	DBA/2.control	4	n/a
	DBA/2.untreated	4	n/a
Open Field paradigm	*Scn1a* +/+ (129) 3 weeks	4	7
	Scn1a +/Δ (129) 3 weeks	7	7
	Scn1a +/+ (129) 6+ months	5	4
	Scn1a +/Δ (129) 6+ months	7	2
	Scn1a +/+.p38γ^{CA} (129) 5-6 months	4	5
	Scn1a +/Δ.p38γ^{CA} (129) 5-6 months	6	4
	Scn1a +/+.control (129) 5-6 months	5	10
	Scn1a +/Δ.control (129) 5-6 months	12	3
	Scn1a +/+ (B6) 3 weeks	7	8
	Scn1a +/Δ (B6) 3 weeks	12	5
	Scn1a +/+ (B6) 2+ months	4	3
	Scn1a +/Δ (B6) 2+ months	5	3
	Scn1a +/+.p38γ^{CA} (B6) 2 months	4	3
	Scn1a +/Δ.p38γ^{CA} (B6) 2 months	5	3
	Scn1a +/+.control (B6) 2 months	13	11
	Scn1a +/Δ.control (B6) 2 months	6	4
	Scn1a +/+.p38γ^{CA} (B6) 5-6 months	6	5
	Scn1a +/Δ.p38γ^{CA} (B6) 5-6 months	6	6
	Scn1a +/+.control (B6) 5-6 months	13	14
	Scn1a +/Δ.control (B6) 5-6 months	4	3

Table S1.

Animal numbers used in survival, EEG and behaviour studies.
